# Attrition of the Midwifery Workforce: Understanding Factors Associated With Leaving the Profession

**DOI:** 10.1111/1475-6773.70143

**Published:** 2026-07-07

**Authors:** E. Brie Thumm, Amy H. Goh, Elizabeth G. Phillips, Zachary Giano

**Affiliations:** ^1^ University of Colorado College of Nursing Aurora Colorado USA; ^2^ Thomas Jefferson University, College of Health Professions, Midwifery & Women's Health Program Philadelphia Pennsylvania USA; ^3^ University of Colorado Center for Innovative Design and Analysis, Colorado School of Public Health Aurora Colorado USA

**Keywords:** burnout, compensation, discrimination, midwife, work environment, workforce, work‐life balance

## Abstract

**Objective:**

To understand why advanced practice midwives (certified nurse‐midwives and certified midwives) leave the workforce.

**Study Setting and Design:**

A multi‐method, multi‐source investigation included secondary analysis of certification data of advanced practice midwives and a cross‐sectional online survey with closed‐ and open‐ended items. Advanced practice midwives' active certification data were used to compare self‐reported demographic characteristics of advanced practice midwives who reported working in the discipline of midwifery to those who did not (*n* = 9704). A survey was conducted in 2022 with individuals who allowed their certification to lapse between 2017 and 2021 (*n* = 303) and individuals who maintained certification but reported not working in the discipline of midwifery (*n* = 1994) to understand why individuals left the workforce and the likelihood of returning. Data were analyzed with ANOVAs, chi‐squares, *t*‐tests, and thematic analysis.

**Data Sources and Analytic Sample:**

Administrative data from the American Midwifery Certification Board (*n* = 9704) and an online survey from a national sample of currently and previously certified nurse‐midwives and certified midwives who had left the workforce (*n* = 646; response rate of 33.8%).

**Principal Findings:**

The most endorsed reasons for leaving were work‐life balance (50.3%, *n* = 325), unsupportive work environment (34.7%, *n* = 224), and schedule (32.0%, *n* = 207). Participants from restrictive regulation states reported lack of opportunities for career advancement, state‐level regulation restricting ability to practice, and lack of employment opportunities at higher rates. Respondents of color more frequently reported inadequate compensation, lack of opportunity for advancement, and workplace discrimination at higher rates. Themes included barriers to re‐entry to practice and compensation.

**Conclusions:**

Work‐life balance and work environment drive midwifery workforce attrition. Individuals of color and those living in states with restrictive regulation face unique challenges. Challenges re‐entering the workforce after leaving create barriers to remaining in the workforce, especially with inequitable compensation.

## Callout Box

- What is known on this topic?
  ○ In the United States, there is a shortage of maternity care providers that contributes to poor and inequitable maternal health outcomes.
  ○ Policymakers see midwives as key to improving maternal health, but the current workforce is too small to meet the needs of birthing individuals.
  ○ Growing the midwifery workforce requires addressing high attrition rates, but little is known about why midwives leave the profession.
- What this study found?
  ○ Work‐life balance, unsupportive work environment, and schedule, specifically on‐call hours and night shifts, drive midwives to leave the profession.
  ○ Midwives from racial and ethnic groups underrepresented in the midwifery profession report lack of opportunity for advancement, low compensation, and workplace discrimination at higher rates than their White counterparts.
  ○ Lack of part‐time employment opportunities and pathways to re‐enter the profession after leaves of absence are barriers to retaining the midwifery workforce.

## 1 Background

Policy makers view the midwifery workforce as key to improving maternal health outcomes and filling gaps in the maternity care workforce; however, the current midwifery workforce is not large enough to meet the needs of birthing individuals in the United States [1, 2]. There are 3.6 million births annually in the United States and only 14,497 advance practice midwives (APMs; certified nurse‐midwives and certified midwives)—4 midwives/1000 births [3, 4]. This contrasts with countries with better maternal health outcomes and an adequate midwifery workforce like Australia where there are 68 midwives for every 1000 births [5]. Given the already small number of midwives, it is essential to retain and maximize the existing workforce. However, a recent study estimated that 30% of APMs were not working clinically 5 years after initial certification and almost half were not working clinically 10 years after initial certification [6]. Developing strategies to retain midwives in the workforce requires understanding why they leave midwifery. In this paper, we report the findings of a study investigating why midwives have withdrawn from the midwifery workforce.

### 1.1 Inadequacy of the United States Maternity Care Provider Workforce Contributes to Inequitable Care

The US maternity care provider workforce suffers from shortages, maldistribution, specialization, reduction of scope, and instability. Further, the maternity care workforce is not equitably distributed across the United States. An estimated 2.3 million women of reproductive age live in maternity care deserts, defined as limited or absent obstetric care facilities or providers [7]. Over a third (39.8%) of US counties do not have a single maternity care provider (APM or obstetrician) and over 50% of counties do not have a birthing facility [8]. There is a shortage of obstetrician‐gynecologists (OB‐GYNs), particularly those who attend births, that is projected to worsen [9]. OB‐GYNs are increasingly specializing in areas of practice that do not include general obstetrics, such as gynecologic oncology or reproductive endocrinology [10]. Rural counties have nearly half the number of obstetricians, and women living in rural areas were attended by midwives 12.5% less often than those in urban areas [7]. Moreover, rural hospitals that do not provide midwifery care are more likely to serve Black, Indigenous, and people of color and Medicaid recipients [11].

### 1.2 The US Midwifery Workforce

Midwifery care in the United States is associated with better outcomes compared to physician care, including decreased cesarean and preterm birth rates [12]; however, the workforce is small, homogeneous, and maldistributed. Understanding the current state of the midwifery workforce and factors that drive attrition requires attention to the historical context shaping the US midwifery profession. In the 19th century there was a robust midwifery workforce that attended half of US births, but by the late 1980s, only 3.7% of US births were attended by midwives [13]. This precipitous decline in midwifery was the consequence of systematic racism and control over female reproduction beginning in the early 20th century [14, 15]. During this time obstetrics emerged as a field of medicine, as birth was medicalized and moved from the home into hospitals to leverage economies of scale, resulting in the monetization of birth [16]. Furthermore, in an act of white supremacy, midwives of color, particularly Black grand midwives, were systematically excluded from attending births through criminalization of midwifery and out‐of‐hospital birth, despite having lower mortality rates compared to physicians [14, 15, 17].

The results of these policies are reflected in the demographic characteristics of the current midwifery workforce and where and how midwives are able to work. The APM workforce is homogeneous (79.8% non‐Hispanic White) and limited in its ability to provide care. After the criminalization of midwifery and out‐of‐hospital birth, it was not until the 1980s that APMs could legally practice in all states and 2007 that they could prescribe in all states [18, 19]. APMs are trained to function as independent clinicians, yet restrictive practice policies limit APMs ability to work independently and are associated with poorer maternal health outcomes [20, 21]. However, in 2025, there are still 18 states that restrict midwives from practicing autonomously by requiring APMs to have physician supervision or written collaborative agreements [20].

### 1.3 Retention as a Barrier to Proposed Expansion of the Midwifery Workforce

Expanding the midwifery workforce requires a two‐pronged approach of increasing the number of entrants into the profession and retaining those already in the profession. While policy makers have taken steps to increase the number of entrants into the profession through funding educational programs, such as the Health Resources and Services Administration's Maternity Care Nursing Workforce Expansion (MatCare) Program (HRSA‐23‐120), there has been less attention to retaining the existing workforce. In 2024, 23.8% of APMs reported not working in the discipline of midwifery when they recertified. Of those not working in midwifery, 7.4% of those reported not working, and 11.4% reported working outside of the discipline of midwifery [4]. In a 2017 national study of the APM workforce, 24.4% reported plans to withdraw from the profession—either by leaving their employer, reducing their hours, or leaving the profession of midwifery altogether [22]. Furthermore, 40.6% of APMs met criteria for burnout, which is associated with turnover [23].

In addition to contributing to workforce shortages, turnover has financial and quality implications that adversely affect patients and systems. While there are a range of estimates in the literature, researchers have estimated that expenditures related to turnover of a primary care physician or nurses can be upwards of $86,000 [24, 25]. Turnover also has negative implications for patient outcomes and healthcare utilization outcomes, such as higher mortality risk 30 days post‐surgery and higher emergency department utilization [26, 27].

Integral to understanding how to retain midwives is understanding why midwives are leaving the profession. While there is literature about midwives' *intention* to leave, there are limited studies of individuals who have left the midwifery profession and none conducted in the United States. To address this gap in the literature, we conducted an investigation of US midwives who reported not working in the discipline of midwifery or allowed their midwifery certification to lapse to understand patterns in their demographic characteristics, why they left the midwifery workforce, and likelihood of returning to the profession.

## 2 Methods

We conducted a multiple method study of individuals who left the midwifery workforce using administrative data and an online survey to (a) characterize individuals leaving the midwifery workforce and (b) identify the reasons why they left.

### 2.1 Participants/Population

The population was individuals who previously worked as APMs but were no longer in the midwifery workforce in any capacity; individuals working in non‐patient‐facing positions in the midwifery field (e.g., education, management) were considered to be in the midwifery workforce. The sample for the secondary analysis of administrative data was individuals who continued to be certified but reported not working in the discipline of midwifery and not being retired at the time of recertification (*n* = 1994). The sample for the survey was comprised of two groups of individuals (*N* = 2997): (1) the sample from the administrative data analysis (individuals who reported not working in the discipline of midwifery and not being retired at the time of recertification [*n* = 1994]) and, (2) individuals who did not re‐certify with AMCB but did not report retirement (allowed their certification to lapse) between 2017 and 2021 (*n* = 303).

### 2.2 Administrative Data Analysis

We analyzed self‐reported administrative data routinely collected by AMCB from APMs at the time of recertification (*n* = 9704). APMs recertify on a 5‐year cycle; therefore, data had been collected on a rolling basis between 2017 and 2021 at the time each participant re‐certified. Age was calculated for all participants in 2021 based on date of birth. The analysis did not include data from initial certification because they are not eligible to join the workforce until after initial certification and therefore could not have left. Age, age at initial certification, race, and ethnicity were compared between those in the workforce and those who had left the workforce using descriptive statistics, ANOVAs, and chi‐square tests.

### 2.3 Survey

A cross‐sectional online survey was sent by AMCB 3 times in October 2022. Participants were asked to answer between 22 and 32 multiple choice and open‐ended questions about reasons for leaving the profession, practice climate of last clinical setting, alternative work since leaving, likelihood of returning, and demographic characteristics (see full description of survey items below and full survey in Supporting Information). Survey data were collected and managed with REDCap [28]. For those who did not recertify, the survey was sent to the most recent email on file. Participants were offered entry into a raffle for one of 30 $100 gift cards.

#### 2.3.1 Survey Items

Reasons for leaving was a multiple‐choice question with 17 response options derived from the extant literature and reviewed with community members prior to survey administration [23, 29, 30, 31, 32]. Participants could select all applicable responses and there was an additional free‐text option. The practice climate of the last clinical setting was measured with the Midwifery Practice Climate Scale, a 10‐item instrument with Likert‐type response options [33]. Likelihood of returning was captured with a single item 5‐point Likert‐type scale with anchors (very likely and very unlikely).

The survey included eight demographic questions, including last state of midwifery employment to determine whether they worked in a state with restrictive or autonomous regulatory environments. At the end of the survey was a single free text item with the statement: Please tell us more about why you are not practicing midwifery or have taken time away from practicing midwifery.

#### 2.3.2 Survey Analysis

Quantitative data were analyzed using descriptive statistics, independent sample *t*‐tests, and chi‐square tests. All quantitative analyses were conducted with SPSS, Version 28.0.1.1. To evaluate for nonresponse bias, we evaluated age, race, and gender of the survey sample relative to the administrative sample of those who were working but outside of the discipline of midwifery and those not working in any capacity. Following quantitative analysis, free text data were analyzed using thematic analysis to corroborate, contradict, and/or expand on quantitative findings [34]. Free‐text responses were reviewed and open coded in Excel independently by two authors (EP and EBT) who then met to discuss how codes coalesced into themes. Finally, a third author (AG) reviewed the data, codes, and themes independently for accuracy and the full team met to refine and contextualize the themes within the quantitative findings. The protocol was approved by the Colorado Institutional Review Board (COMIRB protocol 22‐0839) prior to data collection.

## 3 Results

### 3.1 Administrative Data

Of the 9704 who re‐certified between 2017 and 2021, a total of 2299 (23.7%) reported not working in the midwifery field: 1645 (17.0%) reported working but outside of the discipline of midwifery, 349 (4.6%) reported not working in any capacity, and 305 (3.1%) reported being retired. Those not working or working outside of midwifery were significantly older (*M* = 57.1), compared to those employed in midwifery (*M* = 53.5; *p* < 0.001); however, the difference was less than 4 years. There was not a significant difference in racial or ethnic identities between those working in midwifery and those not working in midwifery (Table 1). Although only 15% of those reporting working outside of midwifery reported where they worked, the most common employer was a university (*n* = 153), while the most common employer of those working within midwifery was private practice (*n* = 3022). Consistently, a significantly larger proportion of those working outside of midwifery held doctorate degrees (21.4%, *p* < 0.01).

**TABLE 1:** Characteristics of AMCB advanced practice midwives^a^ across employment statuses (*N* = 9704).

	Employed as midwife	Employed outside midwifery	Not employed	Retired
	*n* = 7405	*n* = 1645	*n* = 349	*n* = 305
Age, mean (SD)	53.5 (11.3)	57.6 (10.8)	54.8 (11.9)	70.2 (5.5)
Age at first certification, mean (SD)	34.3 (6.7)	34.8 (7.2)	33.4 (6.9)	37.9 (7.9)
Race, No. (%)				
AI/AN, Pacific Islander	38 (0.5)	7 (0.4)	1 (0.3)	0 (0)
Asian	101 (1.4)	29 (1.8)	5 (1.4)	3 (1.0)
Black/African American	424 (5.7)	129 (7.8)	16 (4.6)	17 (5.6)
Hispanic/Latino	329 (4.4)	59 (3.6)	17 (4.9)	8 (2.6)
White/Caucasian	6438 (84.2)	1342 (81.5)	292 (83.7)	263 (86.2)
More than 1 race	52 (0.4)	17 (1.0)	4 (1.1)	1 (0.3)
Choose not to respond/Missing	223 (3.0)	63 (3.8)	14 (4.0)	13 (4.3)

Abbreviations: AI/AN, American Indian/American Native; AMCB, American Midwifery Certification Board; SD, standard deviation.

^a^ Advanced Practice Midwives refers to certified nurse‐midwives and certified midwives.

### 3.2 Survey Results

#### 3.2.1 Sample Characteristics

The survey was sent to 2299 people who met inclusion criteria and had opted into being sent surveys by AMCB (76.7% of sampling frame). Over a third of potential participants responded to the survey (33.8%, *n* = 776). Following case removal for duplicates (*n* = 28) and missing data > 20% (*n* = 96), the final sample was 646 (28.1%). The majority of the sample, 64.6% (*n* = 417), reported being currently certified, while 35.4% (*n* = 229) of the sample had allowed their certification to lapse. The sample was predominantly female, non‐Hispanic, and White. The survey sample showed similar trends to the combined sample of individuals working but outside of the discipline of midwifery and those not working in any capacity from the administrative sample in age (58.4 vs. 57.1), race (81.9% White vs. 87% White), and sex (99% vs. 97.5% female). See Table 2 for characteristics of survey sample.

**TABLE 2:** Characteristics of survey sample (*N* = 646 unless otherwise specified).

	No. (%)
Age, mean	58.4
Years in the profession, mean	16.7
Gender	
Woman	630 (97.5)
Man	3 (0.4)
Trans Female	1 (0.2)
Gender Queer	2 (0.3)
Not reported	10 (1.5)
Sexual orientation	
Straight	582 (90.1)
Gay/Lesbian	13 (2.0)
Bisexual	28 (4.3)
Asexual	2 (0.3)
Other	4 (0.6)
Not reported	17 (2.6)
Race	
Black	31 (4.8)
Asian	12 (1.9)
AI/AN	3 (0.5)
Hispanic	22 (3.4)
White	562 (87.0)
Other	8 (1.2)
Not reported	8 (1.2)
Current midwife certification	
Currently AMCB certified	417 (64.6)
Previously AMCB certified, but did not renew	229 (35.4)
State regulatory environment (*n* = 535)	
Restrictive	262 (49.0)
Autonomous	273 (51.0)
Participants working in healthcare under another licensure (*N* = 179)	
License type	
Adult Health Nurse Practitioner	4 (2.5)
Family Nurse Practitioner	36 (22.1)
Women's health Care Nurse Practitioner	25 (15.3)
Psychiatric Mental Health Nurse Practitioner	17 (10.4)
Pediatric Nurse Practitioner	1 (0.6)
Clinical Nurse Specialists	4 (2.5)
Certified Professional Midwife	0 (0.0)
Registered Nurse	73 (44.8)
Certified lactation Consultant	7 (4.3)
Other	12 (7.4)

Abbreviations: AI/AN, American Indian/American Native; AMCB, American Midwifery Certification Board; SD, standard deviation.

#### 3.2.2 Current Employment

The most common employment positions were working in non‐midwifery healthcare (41.0%, *n* = 265) and not employed (35.8%, *n* = 231); 6.7% (*n* = 43) reported being employed in a non‐healthcare related capacity. Of those working in non‐midwifery healthcare, over half (51.6%, *n* = 163) reported working under another licensure, the most common of which were registered nurse (44.8%, *n* = 73) and family nurse practitioner (22.1%, *n* = 36; see Table 2). Over a quarter of those not providing midwifery clinical care but working in healthcare reported working in education (25.3%, *n* = 80).

#### 3.2.3 Reasons for Leaving

Participants endorsed an average of 3.6 reasons for leaving. The most endorsed reason was work‐life balance (50.3%, *n* = 325). The second and third most endorsed reasons were unsupportive work environment (34.7%, *n* = 224) and schedule (32.0%, *n* = 207). The least commonly endorsed reason for leaving was no longer being interested in the work (0.9%, *n* = 6). People from states with restrictive practice regulation of midwives reported lack of opportunities for career advancement (*c* ^2^ = 8.22, *p* = 0.004), state‐level regulation restricting ability to practice (*c* ^2^ = 14.3, *p* < 0.001), schedule (*c* ^2^ = 4.41, *p* = 0.04), and lack of employment opportunities (*c* ^2^ = 6.16, *p* = 0.01) at higher rates than those in autonomous states.

Given the overall low number of respondents with racial identities other than White, we combined respondents identifying as people of color into a single analytic category due to small cell sizes. Respondents of color more frequently reported inadequate compensation (*c* ^2^ = 5.19, *p* = 0.02), lack of opportunity for advancement (*c* ^2^ = 6.72, *p* = 0.009), and workplace discrimination (*c* ^2^ = 12.58, *p* < 0.001) than White respondents. Sexual minorities were more likely to report being unable to practice consistently with their values (*c* ^2^ = 6.40, *p* = 0.01) and burnout (*c* ^2^ = 10.07, *p* = 0.002), compared to their straight counterparts. Given the skewed number of non‐female respondents, consistent with the population, we were unable to analyze differences by gender identities (see Table 3).

**TABLE 3:** Reasons endorsed for participants' decision to leave midwifery.

	All	State‐level practice regulatory environment		Sig.	Sexual orientation		Sig.	Race		Sig.
		Unrestrictive No. (%)	Restrictive No. (%)		Heterosexual No. (%)	Sexual minority No. (%)		White No. (%)	Non‐White No. (%)	
Inadequate compensation	159 (24.6)	69 (25.3)	73 (27.9)	0.497	140 (24.1)	14 (29.8)	0.380	132 (23.6)	27 (31.4)	**0.020**
Lack of employment opportunity in my area	158 (24.5)	45 (16.5)	66 (25.2)	**0.010**	145 (24.9)	9 (19.1)	0.380	135 (24.1)	23 (26.7)	0.240
Experienced racism and/or discrimination	41 (6.3)	16 (5.9)	17 (6.5)	0.760	35 (6.0)	6 (12.8)	0.070	29 (5.2)	12 (14.0)	**< 0.001**
Work‐life balance	325 (50.3)	134 (49.1)	134 (15.1)	0.630	292 (50.2)	27 (57.4)	0.340	291 (52.0)	34 (39.5)	0.250
Unable to practice according to the MMC	145 (22.4)	64 (23.4)	64 (24.4)	0.790	132 (22.7)	10 (21.3)	0.820	126 (22.5)	19 (22.1)	0.610
Unsupportive work environments	224 (34.7)	108 (39.6)	83 (31.7)	0.060	202 (34.7)	17 (36.2)	0.840	194 (34.6)	30 (34.9)	0.400
Not interested in the work	6 (0.9)	2 (0.7)	2 (0.8)	0.970	4 (0.7)	1 (2.1)	0.280	5 (0.9)	1 (1.2)	0.720
Lack of interprofessional respect	138 (21.4)	61 (22.3)	59 (22.5)	0.960	124 (21.3)	10 (21.3)	0.999	120 (21.4)	18 (20.9)	0.640
Personal health issues	73 (11.3)	37 (13.6)	27 (10.3)	0.250	62 (10.7)	9 (19.1)	0.080	70 (12.5)	3 (3.5)	**0.030**
Burnout	186 (28.8)	96 (35.2)	73 (27.9)	0.070	158 (27.1)	23 (48.9)	**0.001**	166 (29.6)	20 (23.3)	0.560
Schedule	207 (32.0)	97 (35.5)	71 (27.1)	**0.040**	183 (31.4)	19 (40.4)	0.200	181 (32.3)	26 (30.2)	0.730
State‐level reg restrictions	59 (9.1)	12 (4.4)	36 (13.7)	**< 0.001**	50 (8.6)	6 (12.8)	0.330	46 (8.2)	13 (15.1)	**0.010**
Hospital‐level restrictions	78 (12.1)	28 (10.3)	37 (14.1)	0.170	66 (11.3)	6 (12.8)	0.770	65 (11.6)	13 (15.1)	0.170
Issues related to malpractice insurance	49 (7.6)	23 (8.4)	18 (6.9)	0.499	42 (7.2)	5 (10.6)	0.390	42 (7.5)	7 (8.1)	0.590
Unmanageable workload	87 (13.5)	43 (15.8)	34 (13.0)	0.360	74 (12.7)	9 (19.1)	0.210	79 (14.1)	8 (9.3)	0.400
Unable to practice consistently with my values	129 (20.0)	58 (21.2)	54 (20.6)	0.860	109 (18.7)	16 (34.0)	**0.010**	114 (20.4)	15 (17.4)	0.910
Lack of opportunity for career advancement	64 (9.9)	18 (6.6)	37 (14.1)	**0.004**	54 (9.3)	7 (14.9)	0.210	50 (8.9)	14 (16.3)	**0.010**
Other	190 (29.4)	88 (32.2)	75 (28.6)	0.360	171 (29.4)	13 (27.7)	0.800	166 (29.6)	24 (27.9)	0.710

*Note:* Bold indicates statistically significant findings.

Abbreviation: MMC, Midwifery Model of Care.

Almost 35% of respondents reported that they left the midwifery profession because of an unsupportive work environment. The mean score of the Midwifery Practice Climate Scale for the full sample was 25.1, and respondents who reported an unsupportive work environment as a reason for leaving reported a significantly lower score (19.6) compared to those who did not endorse that reason (28.1, *t* = 11.947, *p* < 0.001).

#### 3.2.4 Likelihood of Returning

Almost three quarters (73.7%, *n* = 453) reported they were unlikely to return and only 10.2% (*n* = 63) of respondents endorsed that they were likely or somewhat likely to return. The average age of those who were likely or somewhat likely to return was 52.3 years old, whereas those who were not likely to return were significantly older (61.4 years old, *t* = 8.858, *p* < 0.001). The average years in the profession of those who were likely or somewhat likely to return was 11.9 years, whereas for those who were not likely to return the average was 19.1 years (*t* = 6.957, *p* < 0.001). Table 4 contains likelihood of returning by reasons for leaving.

**TABLE 4:** Likelihood of return by reasons for leaving (*N* = 614).

	Not planning to return	Maybe planning to return	Planning to return
	*n* = 453	*n* = 98	*n* = 63
Inadequate compensation	110 (24.3)	27 (27.6)	16 (25.4)
Lack of employment opportunity in my area	97 (21.4)	31 (31.6)	22 (34.9)
Experienced racism and/or discrimination	26 (5.7)	8 (8.2)	3 (4.8)
Work‐life balance	213 (47.0)	59 (60.2)	32 (50.8)
Unable to practice according to the midwifery model of care	104 (23.0)	23 (23.5)	12 (19.0)
Unsupportive work environments	148 (32.7)	44 (44.9)	17 (27.0)
Not interested in the work	5 (1.1)	1 (1.0)	0 (0.0)
Lack of interprofessional respect	94 (20.8)	25 (25.5)	10 (15.9)
Personal health issues	55 (12.1)	14 (14.3)	3 (4.8)
Burnout	135 (29.8)	28 (28.6)	11 (17.5)
Schedule	141 (31.1)	37 (37.8)	20 (31.7)
State‐level reg restrictions	39 (8.6)	10 (10.2)	7 (11.1)
Hospital‐level restrictions	53 (11.7)	12 (12.2)	10 (15.9)
Issues related to malpractice insurance	35 (7.7)	6 (6.1)	6 (9.5)
Unmanageable workload	55 (12.1)	15 (15.3)	12 (19.0)
Unable to practice consistently with my values	90 (19.9)	23 (23.5)	9 (14.3)
Lack of opportunity for career advancement	44 (9.7)	14 (14.3)	2 (3.2)
Other	140 (30.9)	28 (28.6)	19 (30.2)

*Note:* In all columns, raw numbers are followed by percentage.

#### 3.2.5 Open‐Ended Comment Results

Most participants (77.0%; *n* = 597) left a comment, and the average length of comment was 57 words. Qualitative findings identified reasons for leaving the workforce not found in the quantitative data, expanded on quantitative findings by providing more context, and elucidated patterns of multiple and sequential reasons for withdrawing from the workforce that were not captured in the multiple‐choice format of the quantitative survey.

Three additional reasons for leaving emerged that were not in the multiple‐choice question: lack of part‐time employment opportunity, barriers to re‐entry to practice following time away, and lack of support for transition from education programs to entering the workforce. Barriers to re‐entry to practice focused on feeling like or being told by employers and licensing agencies that they needed clinical re‐training after not practicing for a period. Barriers included not knowing where to get re‐training, availability of re‐training in their geographic region, tuition cost, and opportunity cost (unpaid time required).

Participants provided more detail about work‐life balance and compensation throughout the open‐ended responses. Participants discussed caring responsibilities in their personal lives (e.g., children, aging parents) and having chronic illnesses that were not compatible with the full‐time work and/or being on‐call and overnight shifts. Participants specified compensation was not commensurate with responsibility, stress, and number of hours worked and that they could receive similar (or better) compensation doing non‐midwifery work in healthcare, particularly working as registered nurse. See Table 5 for example quotations of themes identified.

**TABLE 5:** Open‐ended results.

Theme	Quotes
Lack of part‐time employment	
	“I ultimately left to be a stay at home parent as they would not offer my part time to return and I did not want to be away from my baby for a full time position.” (Participant 761)
	“When my children were young, working part‐time in a rural area was not an option for the sole practice in a 200 mile radius. My malpractice insurance would have been the same for 1 delivery or 200. I was told that I needed to bring in a certain amount of ‘income’ to justify my position‐ so I quit to stay at home, and haven't returned to work in a decade. I miss it, however I will never work 60+ hours a week ever again.” (Participant 485)
	“… everywhere I have applied wants me to work an 8–10 h office day which is impossible for me. No one is willing to accommodate a 4–6 h work day. If you want to retain midwives we need to… be more flexible in our work scheduling.” (Participant 215)
Barriers to re‐entry to practice following time away	
	“I left to teach nursing and midwifery for four years, and then couldn't find an organization that would re‐hire me because I hadn't practiced in more than three years. I really had wanted to return.” (Participant 602)
	“Life circumstances occurred and my initial certification lapsed… There were obstacles with reentry related to finding a mentor, then changing regulations for reentry, and what seemed like endless redtape. I eventually gave up, went back to work as and L/D nurse and then into teaching nursing.” (Participant 354)
	“When I attempted to find employment when I no longer need to be home with my children, I was told I would need to update my experience. While that was a reasonable request, it was not possible for me to leave my hometown for several weeks to attend a refresher program.” (Participant 74)
Transition from education programs to entering the workforce	
	“I did not feel adequately prepared by my program and found attending births really stressful in the hospital environment.” (Participant 761)
	“I was unable to find a nurse‐midwife position in my area after graduation despite submitting several applications over the first 5 years after graduation. Having earned a Family Nurse Practitioner Certification as part of a dual MSN degree, I found many more opportunities in that area.” (Participant 545)
	“My first job at an FQHC out of school was really awful and I did not get the nurturing and guidance that a new midwife needs and I feel that affected/stunted my knowledge. I would have a long road to feeling confident again as a midwife if I went back after being gone so long.” (Participant 256)
Compensation	
	“For way more pay and a lot less responsibility I can travel nurse in OB, do what I love and pay the bills. This is hard to compete with. I feel that with all that midwives do‐ the pay should be way more.” (Participant 47)
	“I literally ended an interview when I was told that I would work 4–10s in the clinic for my 40 h, PLUS be expected to cover 10 caLL days a month, 24 h shifts. Wait, you are now expecting me to give up 240 HOURS of my life a month for free???” (Participant 644)
	“Lack of academic credentialling in a university setting despite the expectation that we teach residents and medical students ‐ and despite the fact that I have a PhD. Pay that is less than the night shift nurses make, despite the added education, responsibility, etc.” (Participant 36)
Work‐life balance	
	“There was much joy in the work I did for many years, but much sacrifice in terms of missing special family events and holidays with my kids as well as the sacrifice of health when sleep was eternally compromised. I had to step away to find work life balance.” (Participant 655)
	“After 9 years of practice in a variety of settings, I was never able to find the niche that allowed me to take care of myself. I was always stressed, anxious and in distress for my patients and my colleagues.” (Participant 6)
	“No flexible schedule as a single mother. My employer had daycare and refused to let my child attend the on‐site daycare. I offered to work every Friday, Saturday, and Sunday to accommodate being a single mom and I was told by administration that they could not accommodate my request.” (Participant 255)
	“I was burned out and my adult son who experiences Down Syndrome and my aging/dying parents all lived with me and needed more attention/care from me. Plus Covid hit and I needed to decrease the risks of infection exposure for the vulnerable/at risk family members in my household.” (Participant 348)

Finally, the qualitative data revealed two common patterns of multiple, sequential reasons for leaving the workforce. First, not being able to find part‐time employment following graduation when they wanted to prioritize work‐life balance, then not being able to complete the re‐training needed when they were ready to re‐engage. Second, retiring prematurely to prioritize work‐life balance and not being able to find part‐time employment.

## 4 Discussion

In this sample of APMs we found that work‐life balance coupled with unsupportive work environments and schedule were drivers of leaving the workforce. Our findings show that individuals who have left clinical midwifery were predominantly working as nurses or in academic settings. These settings allow for set schedules and/or schedules that do not require nights and weekends that accommodate work‐life balance, which were consistent with the reasons for leaving indicated in our study. Our finding that the least common reason for leaving the midwifery profession was not being interested in the work, suggesting continued connection to midwifery, was in stark contrast to only 10% reporting they were likely to return. A strong emotional connection to midwifery has been identified in other workforce midwifery studies and may explain why midwives were maintaining their certification despite not planning to return [29, 35]. Individuals of color and those living in states with restrictive regulation face unique challenges that contribute to leaving the workforce. Challenges entering and re‐entering the workforce as work‐life demands ebbed and flowed appeared to create barriers to remaining in the workforce, especially in the context of inequitable compensation.

The reasons for leaving the profession identified in this study were similar to US physicians providing maternity care and midwives in other countries. Among OB‐GYNs in the United States, the reasons cited for specialization (i.e., not practicing as a generalist attending births) have included predictable schedules, less malpractice liability, and larger salaries [36]. Furthermore, OB‐GYNs are choosing to work part‐time or leave clinical practice prematurely to maintain work‐life balance [37]. Family medicine physicians have reported not maintaining their obstetric skills due to not finding employment in settings that provide maternity care and prioritizing work‐life balance [38, 39]. A study of Dutch midwives who had left profession identified conflicts between home and work life, lack of social support from colleagues, value incongruence with their employer, and lack of opportunity for career advancement as reasons for leaving [31]. The Royal College of Midwives in the United Kingdom found similar findings to this study regarding work environment and dissatisfaction with the quality of care they were able to provide driving turnover [40].

Previous studies with US midwives have also demonstrated that unsupportive work environments are associated with the antecedents to attrition (e.g., job dissatisfaction, burnout, and turnover intention) [22, 23, 41]. The findings of this study add to this body of literature, suggesting that unsupportive work environments translate to actual attrition from the workforce. High workload and low staffing have been prominent reasons cited for leaving the midwifery profession in the UK [40]; however, workload was not a leading cause of attrition in this sample of US midwives. This finding is consistent with a previous study of drivers of turnover intention among US midwives that found high workload was secondary to negative practice environment [22].

A distinct difference from OB‐GYNs and international midwives is that the large majority of APMs (99%) also hold nursing licenses, which provides them with alternative employment options. In the context of ongoing nursing shortages and resulting high compensation for nurses, and the flexibility afforded by the shift work model of nursing, it is not surprising that over half of the participants who reported working in healthcare but outside midwifery reported working as registered nurses [1].

The prominence of work‐life balance in our findings suggests that retaining midwives cannot be reduced to focusing on the workplace alone but must account for midwives' experiences outside of the workplace. Midwifery retention and well‐being research has frequently applied the Job Demands‐Resources Model (JDR) that focuses primarily on workplace factors [30, 42, 43, 44, 45]. Our findings suggest that using a model that equally prioritizes non‐workplace factors may be more suitable for studying midwives' attrition. This is consistent with emerging workforce well‐being theory that posits workplace factors cannot be disentangled from the contexts in which workers live, such as the US Centers for Disease Control and Prevention's *Total Worker Health Framework* [46]. Mharapara's and colleagues developed an empirically‐derived contextualized professional well‐being framework based specifically on an integrative review of the midwifery workforce literature [47]. Their framework places midwives' well‐being at the intersection of four equally‐contributing contexts: family and community, work, socio‐cultural, and political‐economic. Future midwifery workforce research will benefit from applying one of these frameworks that explicitly includes aspects of life distinct from work.

Our finding that participants in states with restrictive regulation that limits midwives' autonomous practice left because of lack of opportunities for career advancement, state‐level regulation restricting ability to practice, and lack of employment opportunities helps to provide more insight into workforce studies that have found fewer midwives and lower rates of midwife‐attended births in states with restrictive practice regulation [33, 48]. The findings of this study suggest that there are midwives living in those restrictive states, but their ability to practice is hindered by system‐level factors that lead them to leave the workforce. Given that restrictive practice regulation and low density of midwives—independently and in concert—are associated with adverse pregnancy outcomes (increased primary cesarean and preterm birth rates), this is a key piece of information to inform policy makers about positive workforce implications of removing restrictions on midwifery practice [21].

Our finding that midwives of color reported inadequate compensation, lack of opportunity for advancement, and workplace discrimination more than White respondents is consistent with other health care professions [49, 50, 51]. This suggests midwives of color are particularly vulnerable to attrition due to ongoing intra‐ and inter‐professional racism. Diversifying the current‐day workforce is necessary for amending past harms, as well as a strategy for addressing maternal health racial disparities through racially and culturally concordant care. Recently, there has been formal recognition by the APM professional organization (American College of Nurse‐Midwives) through the *Truth and Reconciliation Resolution* [52]; however, these findings point to ongoing implicit and explicit racism within the profession, as well as within health systems.

Our data suggest that only one in 10 (10.2%) of those who have left are likely to return; therefore, understanding the factors that promote successful return to the workforce is paramount. Successful return to the workforce after leaving because of burnout has been associated with a supportive supervisor and new workplace [53, 54]. Successful return to work after maternity leave, particularly relevant for midwifery because the workforce is 99.0% female and caring for children was a theme in this study, is associated with co‐workers' and supervisors' support, manageable workload, breastfeeding policies, and partner leave [55]. Based on this, cultivating organizational and supervisor expertise in how to support midwives when they return could be a concrete step towards successful return to work for those who return.

There are several regulatory and organizational policy implications for the findings. Organizations can focus on creating job flexibility and part‐time employment to accommodate personal life demands. Investment in *locum tenens* staffing, as seen in several Indian Health Service sites, has the potential to provide flexibility for midwives in both the permanent and temporary roles. Likewise, ensuring midwives have access to medical liability coverage commensurate with the amount of time worked can facilitate part‐time employment. Compensation for APMs must be higher than for registered nurses, reflective of the training and level of responsibility of APMs, and to offset the lack of flexibility of midwifery schedules compared to nursing schedules. The findings suggest that affordable and flexible re‐training programs are a potential strategy to re‐engage individuals who have left the workforce; however, more research is needed because there is limited data about the effectiveness of re‐entry to practice programs for US midwives; international data suggest mixed results of re‐entry programs, with one study finding less than 40% of those completing re‐entry programs actually re‐entered the workforce [56]. Nationally, autonomous practice regulation in all states could reduce barriers to participating in the workforce, such as those reported by respondents in states with restrictive regulation. Additionally, uniform licensure requirements across states and/or licensure portability, such as the Nurse Licensure Compact, would remove barriers to employment when APMs relocate or have opportunities in neighboring states.

Limitations to this study include a convenience sample, which inserted potential response bias by participants who have a strong response, either positive or negative, to leaving the midwifery workforce. Although the demographic characteristics of the survey sample mirrored those of the administrative sample, it should be noted that there may be some degree of nonresponse bias, and that findings may differ slightly from those observed in the full target population, as the analytic sample does not represent a complete census. Potential nonresponse bias could result from disengagement from all professional activities, including surveys, by those who have left the workforce. Another limitation is recall bias because participants could have left the workforce up to 5 years prior to data collection. In this study, we did not aim to isolate the proportion of midwives working in patient‐facing care, which could limit its applicability to workforce projections that are based on patient care demand. Strengths of the study include a response rate of 76.7%, which is well above the typical response rate for nursing studies (10%–15%), and a survey sample that did not differ statistically from the full midwifery workforce [57]. An additional strength is studying actual turnover among midwives instead of its antecedent, *turnover intention*, which is not a consistent or accurate predictor of turnover [58, 59].

## 5 Conclusion

Barriers to retention of the midwifery workforce exist in workplace factors, such as work environment and compensation, but work‐life balance is a driving factor. Groups within the midwifery profession face unique factors that require multi‐level approaches to improving retention, ranging from state‐level policies removing restrictions on practice to eliminating interpersonal racism within the practice settings. There is an opportunity to make returning to the workforce more appealing, with concrete strategies including re‐entry to practice programs and flexible work schedules. Future research will benefit from taking a holistic approach that equally prioritizes the personal and professional in creating strategies to retain the midwifery workforce.

## Funding

This work was supported by the American College of Nurse‐Midwives; Johnson and Johnson Foundation.

## Conflicts of Interest

The authors declare no conflicts of interest.

## Supporting information


**Data S1:** Survey questions.

## Data Availability

The data that support the findings of this study are available from the corresponding author upon reasonable request.
