# Supplementary material for: Attrition of the Midwifery Workforce: Understanding Factors Associated With Leaving the Profession
Source: Health Serv Res. 2026 Jul 7;61(4):e70143. doi: 10.1111/1475-6773.70143 (PMC13338706; doi:10.1111/1475-6773.70143)
Supplement: Supplementary file 1 — Data S1: Survey questions. [file HESR-61-e70143-s001.docx]

**Supplement.** Survey questions

|  | **Question** | **Response Options** |
| --- | --- | --- |
| 1 | What best describes your current certified nurse-midwife/certified midwife status? | 1. Currently AMCB certified 2. Previously AMCB certified, but did not renew my AMCB certification 3. Never certified by AMCB |
| 2 | [If yes to 1(1), 1(2)] What best describes your current employment status? ("employed" includes self-employed) *including women's health/maternal infant/gender-related care | 1. Employed in the field of midwifery* but not providing clinical midwifery 2. Employed in health care but not working within the discipline of midwifery in any capacity 3. [only if yes to 1.1] Employed in clinical midwifery* 4. Employed in a non-health care related field 5. Not employed or not working 6. None of the above |
| 3 | [If yes to 2(6)] Please tell us about your current CNM/CM status | [free text] |
| 4 | [If yes to 2(1), 2(2)] What are of midwifery*/healthcare are you working in? (Check all that apply) *including women’s health/maternal infant/gender-related care | 1. Clinical but practicing under a different certification/licensure 2. Research 3. Education 4. Public Health 5. Policy 6. Healthcare administration 7. Healthcare technology (including FamTech, FemTech) 8. Pharmaceuticals (including cannabis) 9. Other [free text] |
| 5 | [If yes to 4(1)] Which of these certifications/licensures are you practicing under? (Check all that apply) | 1. Adult Health Nurse Practitioner (any type) 2. Family Nurse Practitioner (FNP) 3. Women’s Health Care Nurse Practitioner (WHNP) 4. Psychiatric Mental Health Nurse Practitioner (PMHNP) 5. Pediatric Nurse Practitioner (PNP) 6. Clinical Nurse Specialist (CNS) 7. Certified Professional Midwife (CPM)/Direct-Entry Midwife (DEM) 8. Registered Nurse (RN) 9. Certified Lactation Consultant/IBCLC 10. Other [free text] |
| 6 | [If yes to 2(4)] In what non-healthcare related field are you employed? | [free text] |
| 7 | [If yes to 2(1), 2(2), 2(4), 2(6)] Describe your current job(s)? | [free text] |
| 8 | [If yes to 1(2), 7] Why did you decide not to renew your AMCB certification? | 1. I no longer needed my AMCB midwifery certification to perform my job 2. I did not mean to let my AMCB certification lapse 3. Other [free text] |
| 9 | [If yes to 1(2), 2(1), 2(2), 2(4), 2(5), 2(6)] Have you ever practiced clinical midwifery? | 1. Yes 2. No |
| 10 | [If yes to 9(1)] In what state did you last practice? | 1. Alabama 2. Alaska 3. Arizona 4. Arkansas 5. California 6. Colorado 7. Connecticut 8. Delaware 9. District of Columbia (DC) 10. Florida 11. Georgia 12. Hawaii 13. Idaho 14. Illinois 15. Indiana 16. Iowa 17. Kansas 18. Kentucky 19. Louisiana 20. Maine 21. Maryland 22. Massachusetts 23. Michigan 24. Minnesota 25. Mississippi 26. Missouri 27. Montana 28. Nebraska 29. Nevada 30. New Hampshire 31. New Jersey 32. New Mexico 33. New York 34. North Carolina 35. North Dakota 36. Ohio 37. Oklahoma 38. Oregon 39. Pennsylvania 40. Rhode Island 41. South Carolina 42. South Dakota 43. Tennessee 44. Texas 45. Utah 46. Vermont 47. Virginia 48. Washington 49. West Virginia 50. Wisconsin 51. Wyoming 52. Puerto Rico 53. Other |
| 11 | [If yes to 9(1)] Please describe your last employer (e.g., hospital, FQHC, self-employed in private midwifery practice, self-employed lactation consultant) | [free text] |
| 12 | [If yes to 2(3)] Did you take time away from practicing midwifery? | 1. Yes 2. No |
| 13 | [If yes to 1(2), 2(1), 2(2), 2(4), 2(5), 2(6), 12(1)] What factors affected your decision to no longer, or never, practice midwifery? (Check all that apply) | 1. Inadequate compensation 2. Lack of employment opportunity in my area 3. Experienced racism and/or discrimination in my work as a midwife 4. Work-life balance 5. Unable to practice according to the midwifery model of care 6. Unsupportive work environments 7. Not interested in the work 8. Lack of inter-professional respect 9. Personal health issues 10. Burnout 11. Schedule 12. State-level regulatory restrictions on my ability to practice midwifery 13. Hospital-level restrictions on my ability to practice midwifery 14. Issues related to malpractice insurance 15. Unmanageable workload 16. Unable to practice consistently with my values 17. Lack of opportunity for career advancement 18. Other [free text] |
| 14 | [If yes to 1(2), 2(1), 2(2), 2(4), 2(5), 2(6)] How likely are you to practice clinical midwifery in the future? | 1. Very likely 2. Somewhat likely 3. I don’t know 4. Somewhat unlikely 5. Very unlikely |
| 15 | [If yes to 1(2), 2(1), 2(2), 2(4), 2(5), 2(6)] What would make you begin practicing clinical midwifery (again)? | [free text] |
| 16 | [If yes to 12(1)] What made you return to midwifery? | [free text] |
|  | Section Header:  [If yes to 9(1)] In your last clinical practice setting... "Practice leadership" is defined as the person or persons who have administrative control or other significant influence over your clinical practice, and could include other midwives, physicians, or hospital administrators. | |
| 17 | [If yes to 9(1)] Midwives were represented on important committees in my practice and/or hospital. | 1. Agree 2. Somewhat agree 3. Somewhat disagree 4. Disagree |
| 18 | [If yes to 9(1)] I felt valued in my practice environment. | 1. Agree 2. Somewhat agree 3. Somewhat disagree 4. Disagree |
| 19 | [If yes to 9(1)] Practice leadership was open to midwife ideas to improve patient care. | 1. Agree 2. Somewhat agree 3. Somewhat disagree 4. Disagree |
| 20 | [If yes to 9(1)] I regularly got feedback about my performance from my practice leadership. | 1. Agree 2. Somewhat agree 3. Somewhat disagree 4. Disagree |
| 21 | [If yes to 9(1)] Practice leadership took midwife concerns seriously. | 1. Agree 2. Somewhat agree 3. Somewhat disagree 4. Disagree |
| 22 | [If yes to 9(1)] Practice leadership made efforts to improve working conditions for midwives. | 1. Agree 2. Somewhat agree 3. Somewhat disagree 4. Disagree |
| 23 | [If yes to 9(1)] There was effective communication between midwives and practice leadership. | 1. Agree 2. Somewhat agree 3. Somewhat disagree 4. Disagree |
| 24 | [If yes to 9(1)] My practice environment understood the midwifery model of care. | 1. Agree 2. Somewhat agree 3. Somewhat disagree 4. Disagree |
| 25 | [If yes to 9(1)] My practice environment valued the midwifery model of care. | 1. Agree 2. Somewhat agree 3. Somewhat disagree 4. Disagree |
| 26 | [If yes to 9(1)] Midwifery care was based on a midwifery model of care, rather than a medical model. | 1. Agree 2. Somewhat agree 3. Somewhat disagree 4. Disagree |
| 27 | [If yes to 9(1)] How likely are you to return to practicing clinical midwifery? | 1. Very likely 2. Somewhat likely 3. I don’t know 4. Somewhat unlikely 5. Very unlikely |
| 28 | [If yes to 9(1)] What would make you return to practicing clinical midwifery? | [free text] |
| 29 | [If yes to 1(2), 2(1), 2(2), 2(4), 2(5), 2(6)] Would you be interested in participating in a one-on-one interview to talk about your experience with midwifery? (You will be compensated for your time) | 1. Yes 2. No 3. Maybe |
| 30 | [If yes to 29(1), 29(3)] Please include your email address here: | [free text] |
|  | Section Header:  In order to better understand who is exiting practice, please tell us about yourself | |
| 31 | How many years have you practiced clinical midwifery?(Please provide your lifetime total and round to the nearest year) NOTE: if you never practiced clinical midwifery, enter 0. | [free text] |
| 32 | How many years have you worked in midwifery in any capacity, including clinical and non-clinical work? (Please provide your lifetime total and round to the nearest year) | [free text] |
| 33 | What is your current age? (Please round to the nearest year) | [free text] |
| 34 | What is your racial-ethnic identity? (Check all that apply and provide more specificity)^a^ | 1. Black or African American [free text] 2. Asian [free text] 3. American Indian or Alaskan Native [free text] 4. Native Hawaiian or Other Pacific Islander [free text] 5. Hispanic [free text] 6. White [free text] 7. Other [free text] 8. Not reporting/Unknown [free text] |
| 35 | Did you immigrate to the US from another country? | 1. Yes 2. No |
| 36 | [If yes 35(1)] Where did you immigrate from? | [free text] |
| 37 | What is your gender identify? (Check all that apply) | 1. Woman/Female 2. Man/Male 3. Trans female/Trans woman 4. Trans male/Trans man 5. Genderqueer/Gender non-conforming 6. Gender non-binary 7. Different identity [free text] |
| 38 | What is your sexual orientation? (Check all that apply) | 1. Straight/Heterosexual 2. Gay or Lesbian/Homosexual 3. Bisexual 4. Asexual 5. Different orientation [free text] |
| 39 | Please identify all of your earned academic degrees. NOTE: This question does NOT refer to nursing diplomas or certificates received (such as FNP, or licensure as an RN). | 1. Associate, Nursing 2. Associate, Non Nursing 3. Bachelor’s Nursing 4. Bachelor’s, Non Nursing 5. Master’s, Nursing 6. Master’s, Midwifery 7. Master’s, Not Nursing 8. Master’s, Public Health 9. Doctorate of Nursing Practice (DNP) 10. Doctorate of Midwifery (DM) 11. PhD 12. Other [free text] |
| 40 | Please indicate your current educational debt burden | 1. 25,000 or less 2. 25,001 - 50,000 3. 50,001 - 75,000 4. 75,001 - 100,000 5. 100,001 - 125,000 6. 125,001 - 150,000 7. 150,001 - 175,000 8. 175,001 - 200,000 9. Greater than 200,000 |
| 41 | Do you hold any additional certifications? (Check all that apply) | 1. Adult Health Nurse Practitioner (any type) 2. Family Nurse Practitioner (FNP) 3. Women’s Health Care Nurse Practitioner (WHNP) 4. Psychiatric Mental Health Nurse Practitioner (PMHNP) 5. Pediatric Nurse Practitioner (PNP) 6. Clinical Nurse Specialists (CNS) 7. None (CNM/CM) 8. Other [free text] |
| 42 | Please provide your zip code | [free text] |
| 43 | Please tell us more about why you are not practicing midwifery or have taken time away from practicing midwifery. | [free text] |

^a^Free text option provided for each response option to provide more specificity for racial-ethnic identify.
